# Umbilical cord-derived mesenchymal stem cell extracts reduce colitis in mice by re-polarizing intestinal macrophages

**DOI:** 10.1038/s41598-017-09827-5

**Published:** 2017-08-25

**Authors:** Ji-young Song, Hyo Jeong Kang, Joon Seok Hong, Chong Jai Kim, Jae-Yoon Shim, Christopher W. Lee, Jene Choi

**Affiliations:** 1 Institute for Life Science, University of Ulsan College of Medicine, Asan Medical Center, Seoul, Korea; 2 Department of Pathology, University of Ulsan College of Medicine, Asan Medical Center, Seoul, Korea; 3 Department of Physiology, Asan-Minnesota Institute for Innovating Transplantation, Bio-Medical Institute of Technology, University of Ulsan College of Medicine, Asan Medical Center, Seoul, Korea; 4 Department of Obstetrics and Gynecology, Seoul National University Bundang Hospital, Gyeonggi-do, Korea; 5 Department Obstetrics and Gynecology, University of Ulsan College of Medicine, Asan Medical Center, Seoul, Korea; 6 Department of Molecular and Cellular Biology, University of California, Davis, Davis, California, USA

## Abstract

Human umbilical cord mesenchymal stem cells (hUC-MSCs), originating in Wharton’s jelly, are multipotent stem cells that home to damaged tissues and can modulate the immune system. We examined whether administering extracts of MSCs (MSC-Ex) instead of MSCs could augment the beneficial effects of MSC therapy by overcoming the low homing efficiency of MSCs systemically administered in inflammatory bowel diseases (IBD). Dextran sodium sulfate-induced colitis model was established in C57BL/6 mice, and MSC-Ex was administered intraperitoneally. MSC-Ex reduced colitis, disease activity index (DAI), and histological colitis scores, and increased the body weight. Treatment with MSC-Ex completely blocked the induction of inflammatory cytokines, which were strongly detected in mice with colitis. MSC-Ex shifted the macrophage functional phenotype from M1 to M2 by decreasing the levels of MCP1, CXCL9, and iNOS, but increasing the levels of IL-10, LIGHT, CCL1, and Arg-1. MSC-Ex recovered the destruction of the epithelial barrier in the differentiated Caco-2 cells *in vitro*. Treatment with MSC-Ex was more potent than that with MSC in reducing DAI, the histological score, and nitrite levels. These data strongly support that MSC-Ex treatment can be a potent approach to overcome severe refractory IBD.

## Introduction

Inflammatory bowel disease (IBD) represents a group of pathogenic conditions defined by inflammatory, chronic, and degenerative disorders of the gastrointestinal tract. The main diseases of IBD are Crohn’s disease (CD) and ulcerative colitis (UC). Although the precise etiology of IBD is unknown, it is widely accepted that IBD is caused by a dysregulated immune response to intraluminal commensal antigens in genetically susceptible people^1, 2^.

Professional antigen-presenting cells (APC), including dendritic cells (DC), macrophages (Mϕ), and B-cells, present processed antigens to T cells and shape T-cell-mediated immune responses^3^. Macrophages in the gut have a role in maintaining mucosal homeostasis. In the current paradigm, macrophages are divided into two functional subsets: the classical M1 Mϕ and the alternative M2 Mϕ, although these functional phenotypes are the over-simplified extremes of a broad spectrum of macrophage differentiation states^4, 5^. Microbial stimuli such as lipopolysaccharide (LPS) and immunostimulatory cytokines (IL-12, IL-23, TNFα, and IL6), activate M1 Mϕ, whereas IL-4, IL-13, TGFβ, and IL-10 induce M2 Mϕ. The M1 phenotype is defined by high levels of pro-inflammatory cytokines, reactive nitrogen and oxygen species, and promotion of T helper type 1 (Th1) cell response, resulting in cytotoxicity. In contrast, M2 macrophages are involved in the dampening of inflammation and suppress Th1 responses. The polarization of macrophages is flexible and reversible, to some extent, in concert with T cell specialization (Th1, Th2, and T regulatory cells)^5^.

Stem cell therapies have emerged as novel therapeutic tools for patients with IBD who respond poorly to conventional treatments with anti-inflammatory drugs^6^. In most clinical studies, mesenchymal stem cells (MSCs) are favored over hematopoietic stem cells (HSCs) because of their low immunogenic phenotype^7^. MSCs express defined surface markers, including CD90, CD73, and CD105, and have immuno-regulatory and regenerative properties. They express low levels of type I HLA antigen, but do not express type II HLA antigens or T cell co-activators. MSCs are obtained from the bone marrow, adipose tissue, and the umbilical cord^8^. The numbers of MSCs in the adult bone marrow are very low (0.001~0.01%) and removal of MSCs poses risks for the donor, whereas MSCs from the adipose tissue present a different cytokine profile based on the tissue origin. Human umbilical cord MSCs (hUC-MSCs) are isolated from Wharton’s jelly of the umbilical cord and are distinguishable from the MSCs present in UC blood^9^. hUC-MSCs can be easily isolated in large quantities from postnatal cord tissues, which are usually discarded after birth. Moreover, UC-MSCs can be extensively expanded in culture, frozen/thawed, and possess immunomodulatory properties^10, 11^.

Many studies have shown that the therapeutic capacity of MSCs is mostly mediated by their paracrine effects. MSCs produce a range of cytokines involved in modulating the immune effects^12, 13^. MSCs can suppress T cell proliferation when T cells are stimulated by the pro-inflammatory cytokine INF-γ, and express IL-6 and IL-10 to block macrophage differentiation towards dendritic cells^14^. In mice with induced colitis, MSCs inhibit innate immunity by blocking the differentiation and maturation of monocytes, suppressing T cells, and increasing the populations of T regulatory cells^15^. Recently, Forbes *et al*. have conducted a phase II study using bone marrow derived MSCs. The administration of these MSCs reduced disease activity in patients with luminal CD, which was refractory to biologic therapy^16^. Numerous studies demonstrate that allogeneic MSCs can be a therapeutic alternative for treating patients with IBD. Although MSCs show long-term efficacy, their efficacy in the early stages, for maintaining disease remission, as well as the dose and frequency of infusion, should be determined. To overcome the low homing efficiency and rapid clearance of MSCs post-administration, we investigated the therapeutic effects of UC-MSC extracts (MSC-Ex) in a mouse model of induced colitis. Treatment with MSC-Ex efficiently blocked the induction of pro-inflammatory cytokines and switched the macrophage functional phenotype from M1 to M2 in the colon and peritoneum of mice with induced colitis. The therapeutic effects of MSC-Ex were more potent than those of MSCs.

## Results

### MSC-Ex suppresses dextran sodium sulfate (DSS)-induced colitis

**Figure 1:**
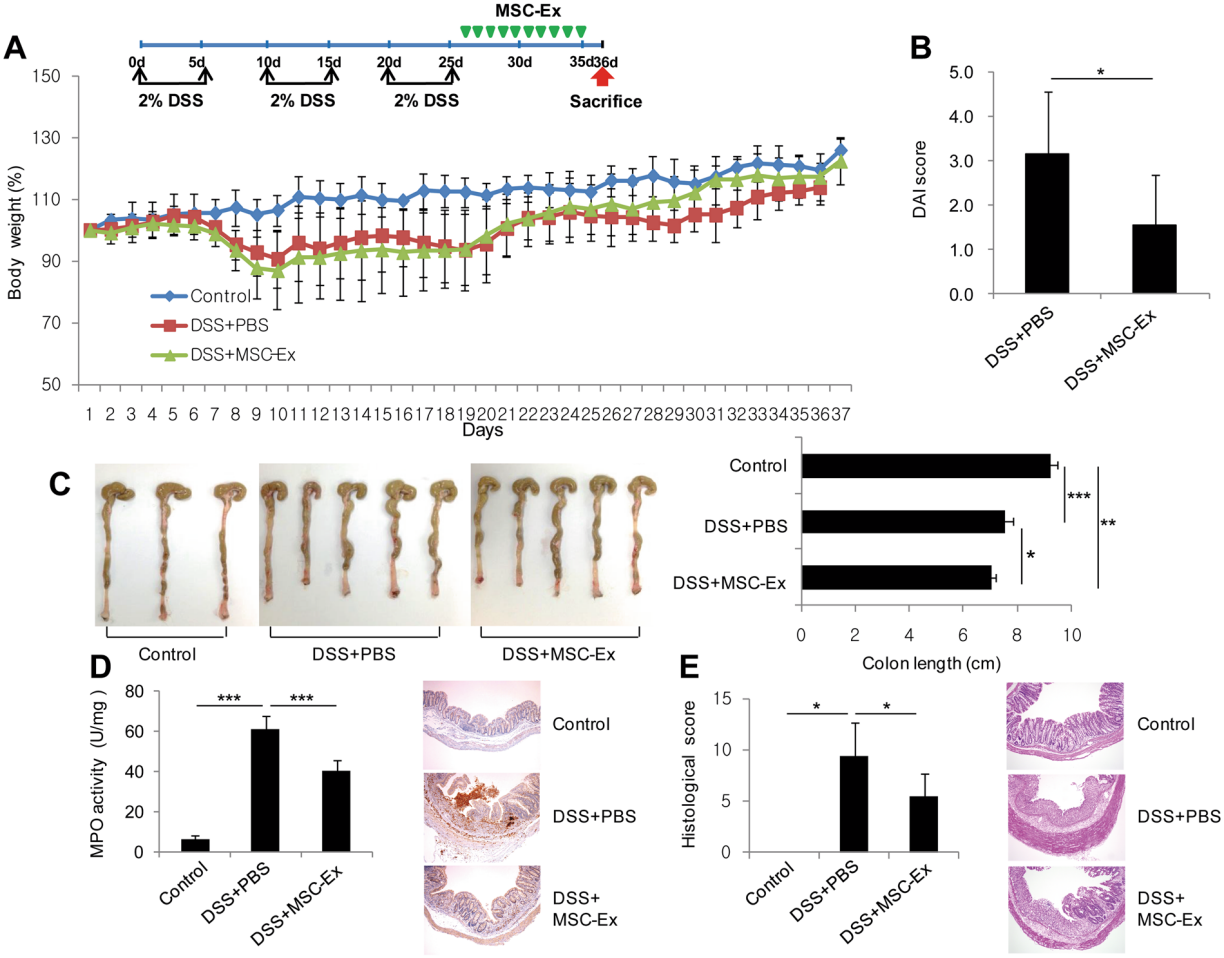
MSC-Ex treatment improves dextran sodium sulfate (DSS)-induced chronic colitis in mice. **(A)** The schematic protocol for DSS-induced mouse colitis and intraperitoneal injection of MSC-Ex (top). Weight loss was measured every day and presented as the percentage change from day 0 (bottom). **(B)** The Disease Activity Index (DAI) scores were measured daily and averaged as the sum of body weight loss, stool consistency, and bloody stool scores. **(C,D)** Colon length **(C)** and the colonic myeloperoxidase (MPO) activity **(D**, left**)** were assessed at necropsy on day 36 following exposure with 2% DSS. The expression of MPO was determined by immunohistochemistry (IHC) analyses of colonic tissues (original magnification, 40x) **(D**, right). **(E**) Colonic damage was assessed by histology using the histological score. Representative hematoxylin and eosin (H&E) stained sections are shown. Data are represented as the means ± s.d. (*n* ≥ *5* mice per group). *P < 0.05, **P < 0.005, ***P < *0.001*.

Although MSCs are an attractive treatment for IBD patients, their survival *in vivo* remains controversial. Many studies have shown that paracrine signaling of MSCs is one of the main mechanisms behind their therapeutic effects^12, 14^. Based on this notion, we hypothesized that a robust administration of paracrine factors, using MSC-Ex, may be a promising therapeutic approach. To assess whether administering MSC-Ex ameliorates ulcerative colitis, we established experimental models of DSS-induced chronic colitis after three cycles of 5-day treatments with 2% DSS and a 5-day recovery between each cycle. The colitis-induced mice showed a loss of ~20% body weight on day 25 (Fig. 1A). The colitis-induced group was intraperitoneally administered MSC-Ex (150 μg per mouse) once per day, for 10 days, on days 26 to 35. The administration of MSC-Ex significantly improved clinical parameters such as body weight and disease activity index (DAI) (Fig. 1A,B); however, the MSC-Ex-treated group did not recover the shortened colon length compared with that of the healthy control group. (7.0 ± 0.2 vs 9.2 ± 0.3 cm) (Fig. 1C). Myeloperoxidase (MPO) activity widely correlates with neutrophil content in the tissue^17^. Compared with the healthy control, MPO activity was increased in the colon of colitis-induced mice. In contrast, the colon from the MSC-Ex treated group showed significantly less MPO activity and immunoreactivity compared with that of the healthy control group (Fig. 1D). The MSC-Ex treated group showed greatly decreased histological colitis scores used to indicate the extent of inflammation, crypt damage, and percentage of colitis. The broken colonic mucosal structure and thickening of the mucosal and muscle layers were greatly recovered in the MSC-EX treated group compared with those in the PBS-treated group (Fig. 1D).

**Figure 2:**
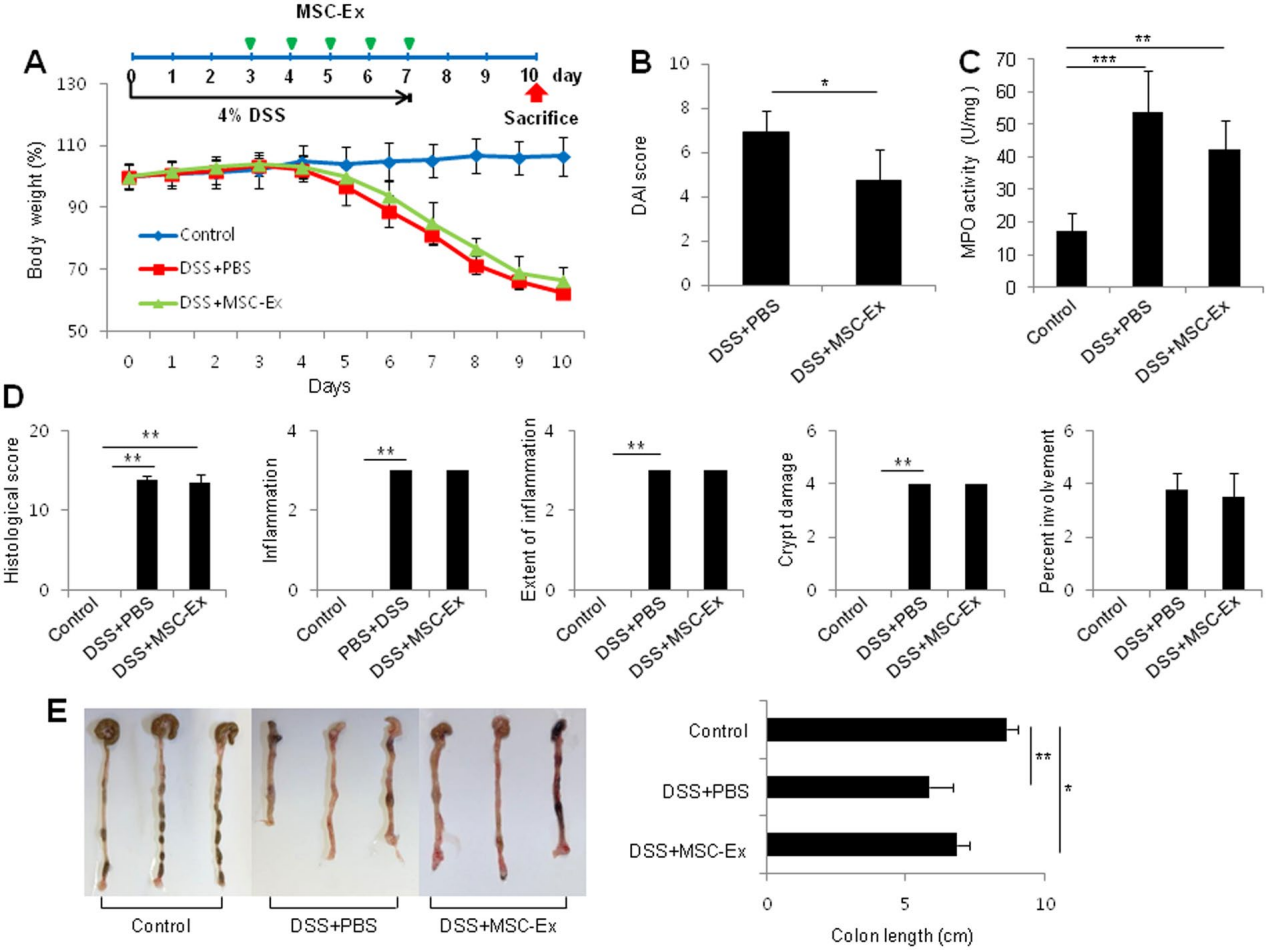
MSC-Ex injection attenuates DSS-induced acute colitis in mice. **(A)** The protocol for DSS-induced colitis and MSC-Ex injection (top). Weight loss was measured every day and expressed as the percentage change from day 0 (bottom). **(B)** The DAI scores were monitored as described in Fig. 1B. (**C**) The colonic MPO activity was measured at necropsy on day 7 following the DSS treatment. **(D)** Histological colitis scores of IBD were assessed by histology. **(E)** Macroscopic appearance (left) and length (right) of the colonic tissues. Data are represented as the means ± s.d. (*n* ≥ *5* mice per group). *P < 0.05, **P < 0.005, ***P < *0.001*.

Because IBD is a chronic intestinal disease, characterized by the prolonged activation of various immune cells in the intestine, immune responses to MSC-Ex may be different in acute DSS-induced colitis. We next used 4% DSS for 7 days to generate acute DSS-induced colitis, and administered MSC-Ex five times on days 3 to 7 (Fig. 2A). We observed a marked decrease in the DAI and MPO activity in the MSC-Ex treated group compared with that in the PBS-treated group (Fig. 2B,C). However, we did not detect differences in the histological colitis score between the DSS-PBS group and the DSS-MSC-Ex group (Fig. 2D). Interestingly, the length of the colon was markedly recovered in the MSC-Ex treated group compared with that in the PBS-treated group (6.8 ± 0.46 vs 5.8 ± 0.87 cm) (Fig. 2E). Collectively, these results indicate that administration of MSC-Ex ameliorates the clinical parameters in DSS-induced colitis.

### MSC-Ex reduced pro-inflammatory cytokines in the intestine

**Figure 3:**
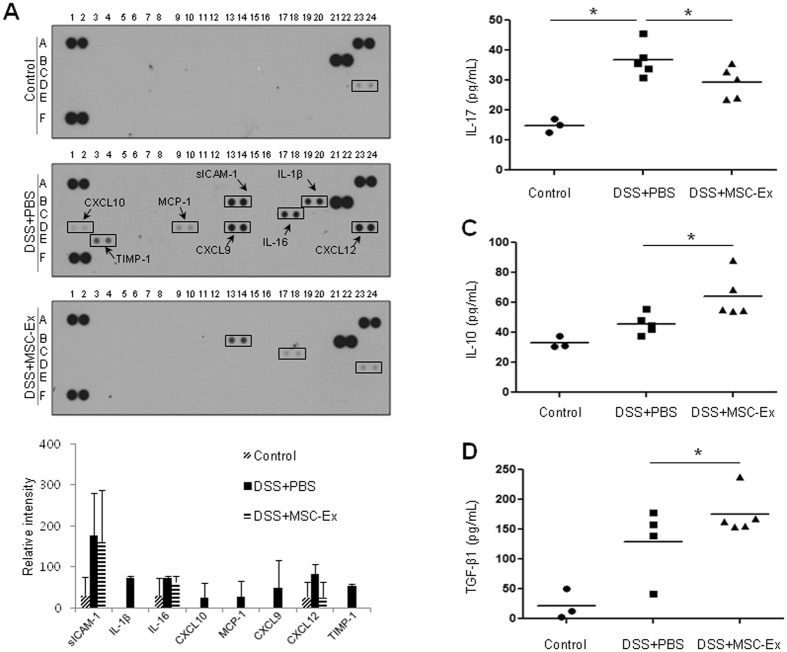
MSC-Ex decreases the expression of pro-inflammatory cytokines in chronic DSS-induced colitis. **(A)** The levels of inflammation-related cytokines were analyzed using colonic tissues by immunoblot analysis (top). The relative expression of each cytokine was quantified (bottom); data representative of those obtained in three independent experiments are shown. The levels of IL-17 **(B)**, IL-10 **(C)**, and TGF-β1 **(D)** in colon extracts were measured by ELISA in the colons of chronic colitis-induced mice at necropsy on day 36 following DSS exposure. Data are represented as the means ± s.d. (*n ≥ 5*). *P < 0.05, **P < 0.005, ***P < *0.001*.

To examine the immune response modulation of MSC-Ex, we analyzed cytokine expression in the colon tissues of chronic colitis-induced mice. Whole cell lysates, prepared from the colonic tissues, were analyzed by immunoblotting using the human cytokine antibody array. Cytokine protein arrays showed that the levels of several cytokines, including sICAM-1, IL-1β, IL-16, CXCL10, MCP-1, CXCL9, and TIMP-1, were upregulated (≥50-fold) in the colitis-induced PBS-treated group compared with those in the normal healthy control group. In contrast, the colons of the colitis-induced MSC-Ex-treated group showed greatly reduced levels of these cytokines. The cytokine profile of the MSC-Ex treated group was similar to that of the healthy control group on the background of inflammatory environment (Fig. 3A). We further analyzed the expression of the cytokines IL-17, IL-10, and TGF-β1 using enzyme-linked immunosorbent assay (ELISA) assays. The level of the pro-inflammatory cytokine IL-17 was significantly decreased (Fig. 3B), whereas those of the anti-inflammatory cytokines IL-10 and TGF-β1 were increased in the MSC-Ex-treated group compared with those in the PBS-treated group (Fig. 3C,D). These results indicate that MSC-Ex markedly reduces the inflammatory state in the colitis-induced mice.

### MSC-Ex shifted the macrophage functional phenotype in the colitis-induced colon

**Figure 4:**
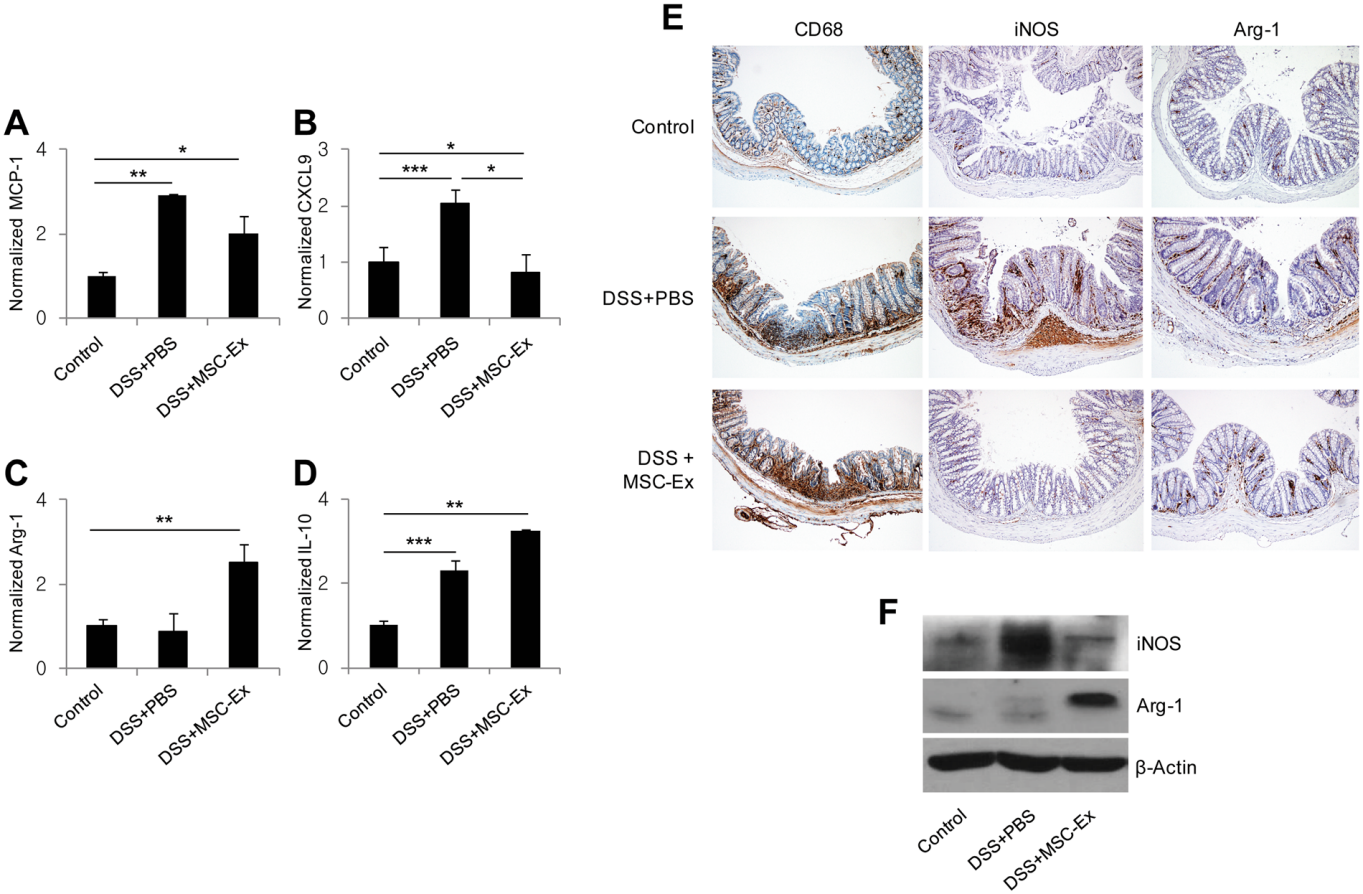
MSC-Ex shifted the macrophage phenotype from M1 to M2 in the colon of mice with chronic colitis. The relative expressions of the macrophage phenotype-associated genes were evaluated by qRT-PCR: **(A,B)** MCP-1 and CXCL9 (M1 marker), **(C,D)** Arg-1 and IL-10 (M2 marker). **(E)** Representative photomicrographs of CD68 (macrophage marker), iNOS (M1 marker), and Arg-1 (M2 marker), immunohistochemically labeled in the colonic tissues of colitis mice. The photomicrographs were acquired at 100x magnification. **(F)** The protein levels of intracellular iNOS and Arg-1 were evaluated by western blotting. Data are represented as the means ± s.d. (*n ≥ 3*). *P < 0.05, **P < 0.005, ***P < *0.001*.

To investigate the mechanism by which MSC-Ex exert their therapeutic effects, we next focused on the colonic macrophages because they are one of the most numerous leukocytes, and play a key role in homeostasis through the M1/M2 polarization in the colon^5^. We isolated RNA and examined the expression levels of inflammatory markers using quantitative real-time RT-PCR (qRT-PCR) from the digested colon tissues of chronic colitis-induced mice. The levels of M1 markers, such as MCP-1 and CXCL9, were greatly decreased (Fig. 4A,B), while those of the M2 markers, such as arginase-1 (Arg-1) and IL-10, were significantly increased in the mice treated with MSC-Ex (Fig. 4C,D). M1 and M2 macrophages have marked differences in arginine metabolism. M1 macrophages metabolize arginine to nitric oxide via the inducible nitric oxide synthase (iNOS), while the M2 macrophages metabolize arginine to ornithine via Arg-1^18^. To better define the macrophage phenotypes recruited in colitis, the colons were stained with anti-CD68, a macrophage-specific marker, as well as with anti-iNOS and anti-Arg-1^5^. Increased infiltration of CD68-positive macrophages was detected in the inflamed mucosa of the colitis-induced mice. The iNOS-positive cell infiltrations were predominantly observed in the colitis-induced PBS-treated group compared with the healthy control and colitis-induced MSC-Ex treated groups. Conversely, Arg-1 positive cells were prevalent in the MSC-Ex-treated group, even though inflammation was nearly ameliorated (Fig. 4E). These results were consistently observed in immunoblot analysis, which showed decreased levels of iNOS, and increased levels of the Arg-1 proteins, in MSC-Ex-treated colons (Fig. 4F). These results imply that MSC-Ex shifts the macrophage phenotype to exert therapeutic outcomes.

### Phenotype switch of peritoneal macrophages by treatment with MSC-Ex

**Figure 5:**
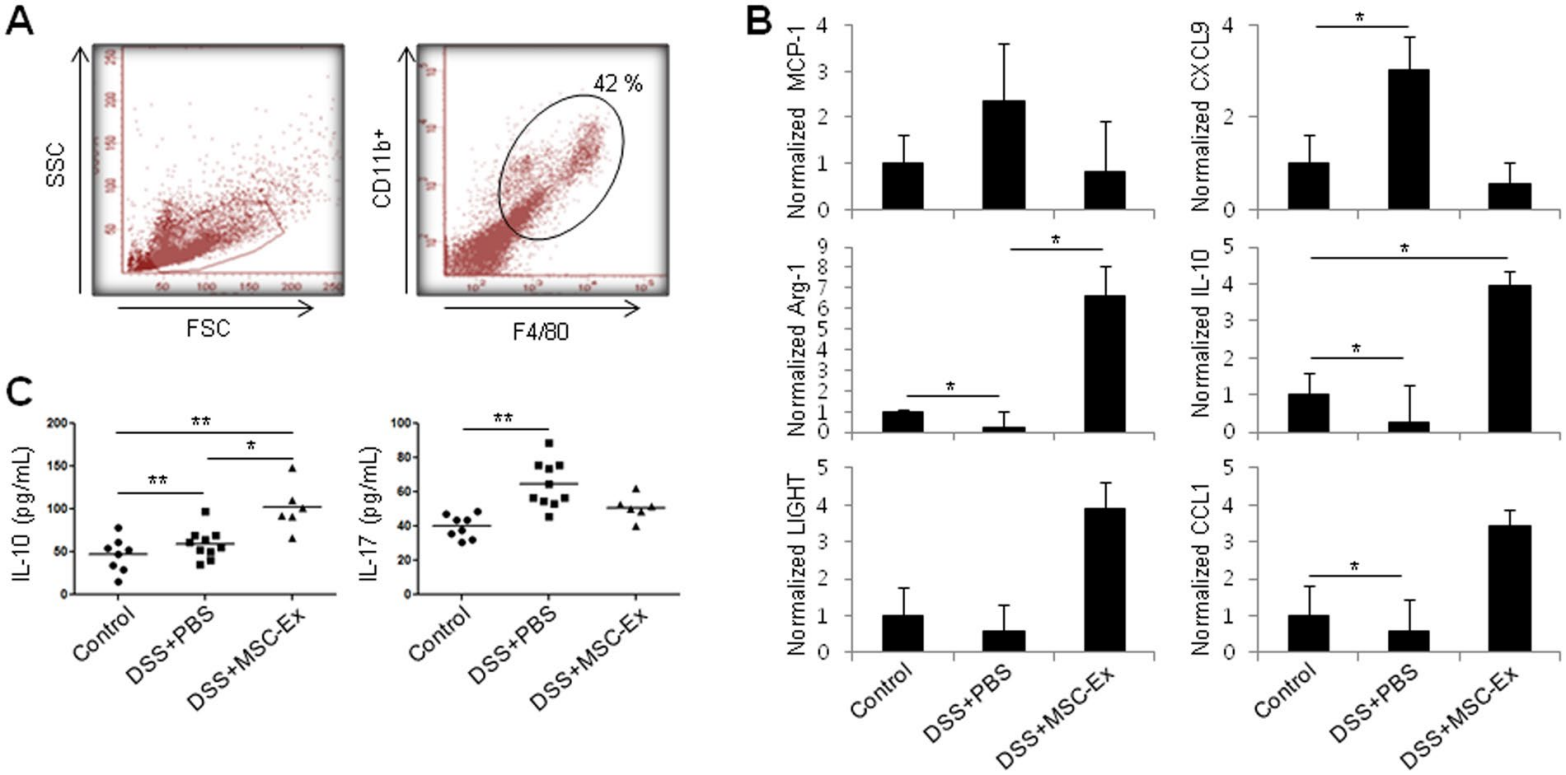
Phenotype shift of peritoneal macrophages by MSC-Ex. **(A)** F4/80^+^/CD11b^+^ macrophage cells were isolated by flow cytometry from the peritoneum of acute colitis-induced mice. **(B)** The relative expressions of MCP-1 (M1 marker), CXCL9 (M1 marker), Arg-1 (M2 marker), IL-10 (M2 marker), LIGHT (M2 marker), and CCL1 (M2 marker) in F4/80^+^/CD11b^+^ peritoneal macrophage cells were examined by qRT-PCR analysis. **(C)** IL-17 (left) and IL-10 (right) levels in the colonic tissues were determined by ELISA. Data are represented as the means ± s.d. (*n > 3*). *P < 0.05, **P < 0.005, ***P < *0.001*.

Large peritoneal macrophages (F4/80^high^ and CD11^high^) are maintained in the peritoneal cavity, can undergo rapid proliferation, and arrive at the afflicted inflammatory tissues^19^. We investigated whether MSC-Ex treatment influences the peritoneal macrophage phenotype. F4/80^+^/CD11b^+^ macrophage cells, from the peritoneum of mice with acute colitis, were isolated by flow cytometry and the M1/M2 status was analyzed by qRT-PCR (Fig. 5A). M1 markers of MCP1 and CXCL9 were decreased, while M2 markers of Arg-1, IL-10, LIGHT, and CCL1 were increased in the MSC-Ex treated group compared with the PBS-treated group (Fig. 5B)^5^. We further measured the levels of IL-17 and IL-10 in the macrophages using ELISA. The level of anti-inflammatory cytokine IL-10 was highly enhanced, however, that of the pro-inflammatory cytokine IL-17 was reduced in the MSC-Ex treated group (Fig. 5C). These results confirmed that MSC-Ex modulates macrophage polarization from the M1 to M2 state in colitis-induced mice.

### MSC-Ex recovered damaged intestinal epithelial barrier

**Figure 6:**
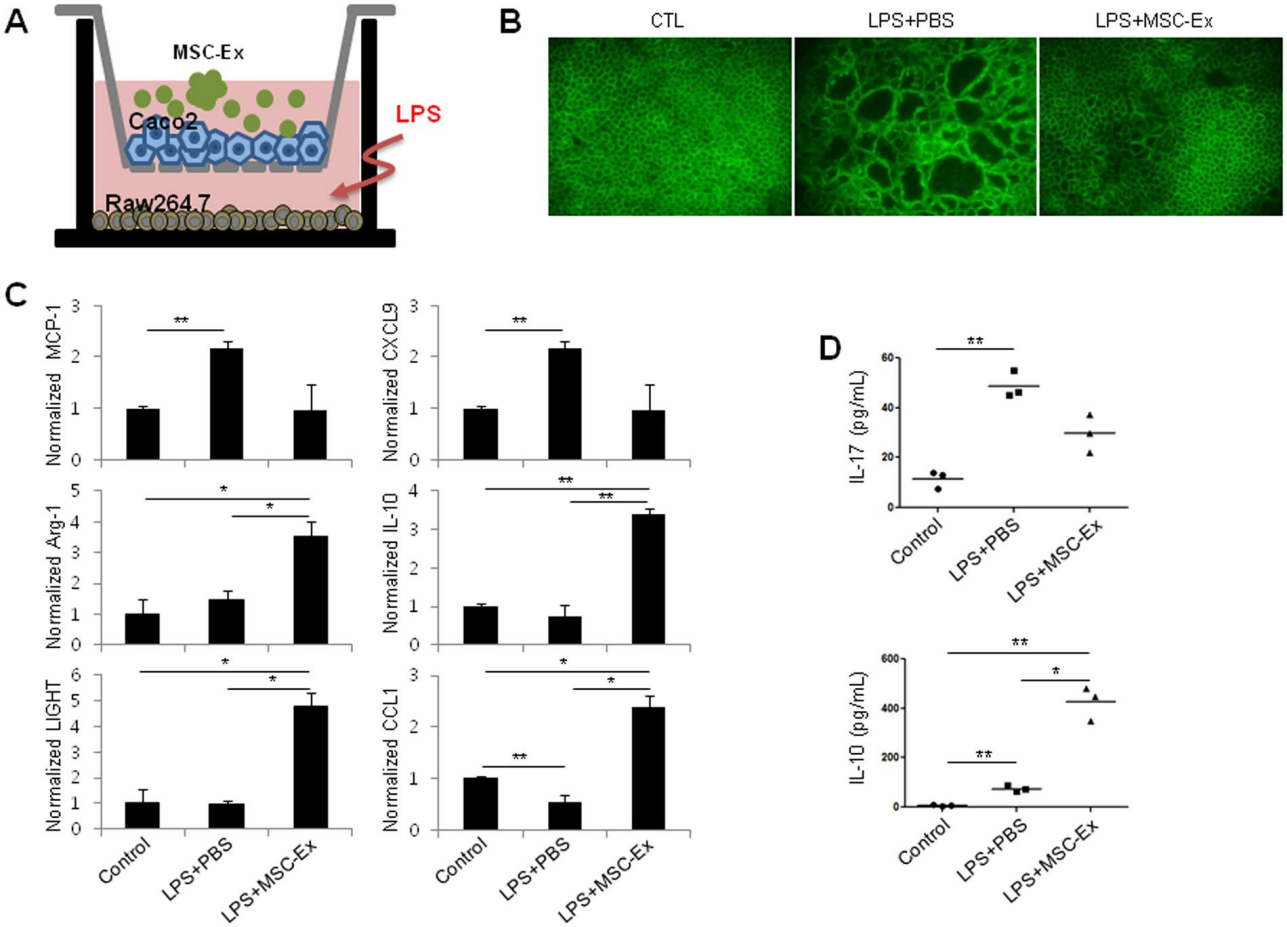
MSC-Ex protects intestinal adherens junctions in an *in vitro* model. **(A)** Schematic representation of the *in vitro* co-culture system. Caco-2 cells were cultured on the upper transwell inserts in multiple well plates containing RAW 264.7 cells. To imitate inflammation in the gut, LPS was added to the basolateral compartment, and MSC-Ex was administered to the apical side. **(B)** Immunofluorescent staining for E-cadherin using FITC-conjugated antibodies. Continued epithelial barrier, indicated by E-cadherin staining, is disrupted in the absence of MSC-Ex. Magnification, 40x. **(C)** The transcripts of MCP-1 (M1 marker), CXCL9 (M1 marker), Arg-1(M2 marker), IL-10 (M2 marker), LIGHT (M2 marker), and CCL1 (M2 marker) in RAW 264.7 cells were examined by qRT-PCR analysis after 4 h of LPS (1 μg/mL) treatment and subsequent MSC-Ex (30 μg/mL) administration for 24 h. **(D)** The protein levels of IL-17 (top) and IL-10 (bottom) were measured by ELISA using culture supernatants from the basolateral side. Data are represented as the means ± s.d. (*n > 3*). *P < 0.05, **P < 0.005, ***P < *0.001*.

The disruption of the intestinal mucosal barrier is an important indicator in the pathogenesis of IBD. The permeability of the intestinal epithelial barrier is regulated by the adhesion structures of adherens and tight junctions^20^. Adherens junctions, comprised of E-cadherin and nectins, are responsible for initiating cell-cell contacts. To investigate whether MSC-Ex treatment generated the functional recovery of the gut barrier, we developed a co-culture system of intestinal epithelial Caco-2 cells with RAW 264.7 macrophage cells as an intestinal model *in vitro* (Fig. 6A). After 14 days of culture, Caco-2 cells underwent spontaneous differentiation, leading to the formation of a monolayer with features similar to those of mature enterocytes. We observed a pattern of continuous E-cadherin staining in the monolayer of Caco-2 cells. LPS treatment induced inter-epithelial gaps and destabilization of cell-cell junctions in the differentiated monolayer sheet of Caco-2 cells, mimicking the destruction of the epithelial barrier. Co-administering the MSC-Ex (30 ug/mL) treatment with LPS greatly prevented the destruction of the epithelial barrier (Fig. 6B). Gene expression analysis of the co-cultured RAW 264.7 cells indicated that the expressions of the M1 markers (MCP-1 and CXCL9) were reduced, while those of the M2 markers (Arg-1, IL-10, LIGHT, and CCL1) were increased (Fig. 6C). In agreement with the above results, the expression of IL-17 was reduced, while that of IL-10 was increased, in the RAW 264.7 culture media (Fig. 6D). These results indicate that MSC-Ex exerts therapeutic effects by inhibiting the M1 and stimulating the M2 phenotype, resulting in the protection of adherens junctions and epithelial integrity in the colon.

### MSC-Ex is superior to UC-MSCs in chronic IBD models

**Figure 7:**
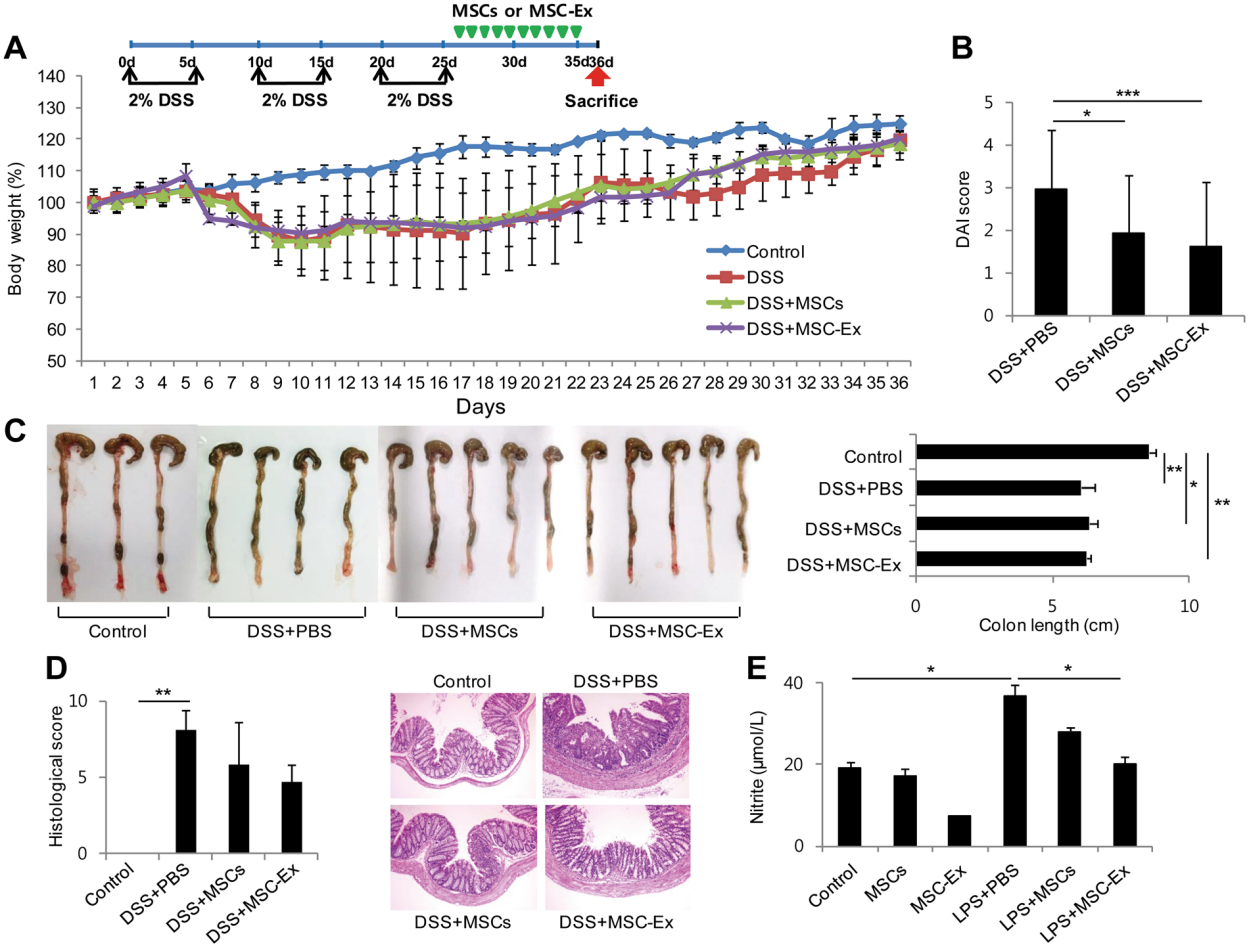
MSC-Ex is more efficient than MSCs in the chronic colitis model. **(A)** The schematic protocol for chronic DSS-induced mouse colitis and intraperitoneal injection of MSC-Ex as described in Fig. 1A. (**B**) DAI was determined as described in Fig. 1B. (**C**) Colon length. **(D)** Histological scores were calculated as described in Fig. 1E. Representative H&E stained sections are shown. **(E)** The levels of nitrite, a final metabolite of NOS, were measured using the culture media from the basolateral side in the co-culture system of Caco-2 and RAW 264.7 cells. Data are represented as the means ± s.d. (*n ≥ 5* mice per group). *P < 0.05, **P < 0.005, ***P < *0.001*.

To strengthen the evidence for the therapeutic effects of MSC-Ex, we compared the therapeutic efficacy between MSC-Ex and MSCs in chronic IBD models. MSCs and MSC-Ex, prepared from an identical number of MSCs (1 × 10^6^ cells), were administrated intraperitoneally in colitis-induced mice (Fig. 1A). Both treatments significantly ameliorated colitis; however, the DAI and histologic colitis scores were lower in the MSC-Ex-treated group compared with those in the MSC-treated group in the absence of differences in the colon length (Fig. 7B–D). In the co-cultured RAW 264.7 cells, MSC-Ex significantly reduced the levels of nitrite compared with those in the MSC-treated cells, in the absence and presence of LPS stimulation (Fig. 7E); nitrite is a major final metabolite of NOS used as a marker of inflammation in IBD. These results strongly support our theory that treatment with MSC-Ex is more efficient than that with MSCs in inducing immunosuppression.

## Discussion

MSC therapy has emerged as a promising therapeutic strategy for inflammatory bowel disease. MSC therapy for IBD is well tolerated, but its clinical results with respect to efficacy are inconsistent, possibly because of the different methods used for the isolation and expansion, and MSC types such as bone marrow-, adipose- or umbilical cord-derived MSCs^15, 20–23^. The immuno-modulatory ability of MSCs may be the result of their ability to migrate to inflamed tissue. However, emerging data show that only a limited number of engrafted MSCs reach the inflamed colon, and that the immunosuppressive activity of MSCs is mainly mediated by soluble paracrine factors^24^. Here, we focused on evaluating the therapeutic effects of umbilical cord-derived MSC-Ex and the mechanisms underlying the immunosuppressive activities of MSC-Ex in DSS-induced colitis.

Our results show that intraperitoneally injected MSC-Ex significantly improved the inflammatory symptoms in DSS-induced IBD mice. UC-MSCs can produce components that interfere with both the innate and adaptive immune systems. UC-MSCs produce IL-6, required by the dendritic cells to acquire tolerogenic phenotypes, prostaglandin E2 (PGE2), which inhibits the cytotoxicity of NK cells and T cell proliferation, and indoleamine 2,3-dioxygenease (IDO), which represses the differentiation of circulating T follicular helper cells; UC-MSCs also stimulate immunomodulatory cytokines such as TGFβ1 and TNF-stimulated gene 6 proteins (TSG-6)^25–28^. The intraperitoneal administration of MSC-Ex, used in our study, robustly induced the influx of anti-inflammatory factors to the highly inflamed peritoneum of DSS-colitis induced mice. Antibody arrays showed that the cytokine profiles of colonic extracts from MCS-Ex-treated colitis mice were similar to those from normal healthy mice (Fig. 3A). The expressions of ICAM-1, IL-1β, IL-16, CXCL10, MCP-1, CXCL9, CXCL12, and TIMP-1 were greatly reduced in MSC-EX-treated colitis group compared with those in the PBS-treated colitis group. CXCL9, CXCL10, CXCL12, IL-16, and MCP1 are chemo-attractants that recruit monocytes, T cells, and dendritic cells to the inflammatory site; the expression of these cytokines is significantly increased in IBD patients^29, 30^. Thus, MSC-Ex contributes to a counter-regulatory response to excessive pro-inflammatory cytokine activity in the colitic colon.

Macrophages are divided into two functional subsets: the classically activated M1 (pro-inflammatory M1) and alternatively activated M2 (anti-inflammatory M2) macrophages^31^. MSC-Ex altered the macrophage phenotype from M1 to M2 by significantly increasing the expression of Arg-1, LIGHT, and CCL1 in the peritoneal macrophages (Fig. 5). The altered macrophage signature was detected in mRNA and protein in both acute and chronic colitis models. Treatment with MSC-Ex, administered to RAW 264.7 macrophages co-cultured with intestinal epithelial Caco-2 cells, led to a distinct shift from the M1 to the M2 phenotype, preserving the epithelial barrier function (Fig. 6). The ratio of iNOS (M1-specific enzyme) to Arg-1 (M2-specific enzyme), indicative of arginine metabolism, was radically decreased in the MSC-Ex-treated colon of chronic colitis-induced mice (Fig. 4E,F). Consistent with our findings, other groups have shown that intraperitoneally injected M2 macrophages enter the circulation, extravasate into the colon, and decrease inflammation, resulting in the scavenging of debris, angiogenesis, and tissue repair. M2 macrophages recruit Th2 lymphocytes, eosinophils, basophils, and Tregs using CCL17, CCL22, and CCL24^30, 32^. Thus, the M2 polarization of macrophages by MSC-Ex may result in a favorable environment that accommodates tissue homeostasis and repair.

IL-10 plays a major role in the maintenance of intestinal homeostasis. IL10^−/−^ mice develop spontaneous colitis^33^. Patients with a gene mutation in the IL-10 receptor (IL-10R) develop severe pediatric IBD^34^. In our study, MSC-Ex-treated colonic tissues and peritoneal macrophages produced markedly high levels of IL-10 transcripts. Leung and colleagues^35^ reported that bone marrow-derived M2 macrophages from wild type mice reduced dinitrobenzene sulfonic acid (DNBS)-driven colitis more than the M2 macrophages from IL-10^−/−^ mice. Notably, all macrophages synthesize IL-10; however, the M2 macrophages produce less nitric oxide. IL-10, secreted by the M2 macrophages, may inhibit pro-inflammatory cytokines. The anti-colitic effect of MSC-Ex may be dependent in part on the capacity to stimulate IL-10 production in macrophages.

In IBD, the rationale for MSC therapy is mainly centered on their immunosuppressive activities. MSC cells have been a source of therapeutic tools because of their sustained and slow release of IL-10, in response to the intestinal environment, and trafficking to the damaged colon. We demonstrate that re-polarization of differentiated macrophages by MSC-Ex re-educated macrophages to continuously secret IL-10. In this study, we acknowledge that the therapeutic components of MSC-Ex were not fully characterized. However, we found that MSC-derived conditioned medium (MSC-CM) and MSC-Ex have very distinct cytokine profiles, and that the MSC-Ex prepared from different umbilical cords show consistent cytokine expression patterns (Supplementary Figure 1). In addition, MSC-Ex-mediated therapeutic effects were not observed with extracts from WI-38 fibroblasts (Supplementary Figure 2). The data support the specificity of MSC-Ex for IBD treatment. We also observed local immunologic responses at the site of MSC injection, but not at that of MSC-Ex injection, in the colitis-induced mice (unpublished data). Although further studies are required to determine whether treatment with MSC-Ex can be used safely to treat patients with IBD, our findings suggest that MSC-Ex could provide an alternative therapeutic tool to overcome the low homing efficiency of MSCs in stem cell therapies for IBD patients.

## Materials and Methods

### Ethics statement

This work was approved by the Institutional Review Board of Asan Medical Center (authorization no. 2015–0303). All women provided written informed consent. The research was conducted in accordance with Helsinki Declaration. All animal experiments were performed in accordance with relevant guidelines and regulations of Animal Ethics Committee of Asan Medical Center (authorization no. 2015-12-220) accredited for laboratory animal care by Ministry of Food and Drug Safety of South Korea.

### Isolation and expansion of UC-MSCs

The umbilical cord samples were obtained at the time of caesarean delivery from healthy pregnant women. UC-MSCs were prepared according to the method previously described^36^. The cord was rinsed several times with sterile saline and cut into 3-mm-thick pieces. The vessels and amnion were removed from cord segments. Wharton’s jelly tissues were minced and digested for 3 h in MEM (11095-080; Invitrogen-Gibco, Carlsbad, CA) with 0.1% collagenase A (10103578001; Roche, Mannheim, Germany) at 37 °C in a shaking incubator. To remove large pieces of tissue, the cells were filtered through a 70 μM mesh (103760; BD Falcon, San Jose, CA) and pelleted using low-speed centrifugation at 200 × *g* for 10 min. The isolated cells were plated in DMEM supplemented with 10% FBS, 50 μg/ml penicillin and 100 μg/ml streptomycin (Invitrogen-Gibco) at 37 °C in a humidified 5% CO_2_ incubator. MSCs were selected using attachment to plastic culture plates after 72 h. MSCs at passages 3, 4, and 5 were pooled for MSC-Ex or MSC therapy.

### Preparation of MSC-Ex

UC-MSCs (1 × 10^6^ cells) were washed twice with cold phosphate-buffered saline (PBS) and resuspended in five times the packed cell volume of PBS. After 30 min of incubation on dry ice, the cells were transferred to a water bath maintained at 37 °C for 3 min. The freezing and thawing was repeated three times. After centrifuging at 12,000 × *g* for 10 min, the supernatant was collected and stored at −80 °C.

### DSS-induced colitis model

To generate the model of acute colitis, 6-week-old C57BL/6 mice were treated with 4% DSS (160110; MP Biomedicals, Solon, OH) in drinking water from day 0 until day 7. The mice were divided into two groups of 7 mice per group. The mice were treated with MSC-Ex (150 ug/mouse) in 100 μL saline or PBS by intraperitoneal injection, for 5 days, starting on day 3. For the chronic colitis model, the mice were treated with 2% DSS in drinking water for 5 days of the three cycles (5-day DSS treatments on days 0~5, 10~15, and 20~25, followed by a 5-day recovery period after each treatment). On day 26, the mice were intraperitoneally treated with PBS or MSC-Ex (150 ug/mouse) for 10 days. The disease activity index (DAI) represents the combined scores of weight loss, stool consistency, and presence of blood in the feces and anus. DAI was assessed daily as described previously^35^: body weight loss (0, none; 1, 1–10%, 2, 11–20%), stool consistency (0, normal; 1, soft; 2, liquid), and the presence of blood (0, negative fecal occult bleeding; 1, positive fecal occult bleeding; 2, visible fecal occult bleeding). The score of each parameter was summed up from the first day of MSC-Ex injection to the day of sacrifice, and the summed score was averaged to yield the final score.

### Histological evaluation and immunohistochemistry (IHC)

Colon sections were stained with hematoxylin and eosin (H&E) and graded for inflammation (0, none; 1, slight; 2, moderate; 3, severe), extent (0, none; 1, mucosa; 2, mucosa and submucosa; 3, transmural), crypt damage (0, none; 1, basal 1/3 damage; 2, basal 2/3 damage; 3, only surface epithelium lost; 4, entire crypt and epithelium lost), and percent of involvement (1, 1~25%; 2, 26~50%; 3, 51~75%; 4, 76~100%) as described^37^. The score of each parameter was multiplied by a factor reflecting the percentage of tissue involvement to yield the final score. For evaluation using IHC, 5-μm-thick tissue sections were transferred onto adhesive slides and dried at 62 °C for 30 min. After incubation in 3% H_2_O_2_ to block the endogenous peroxidase activity, tissue sections were subjected to standard heat-mediated epitope retrieval in ethylene diamine tetraacetic acid (pH 8.0) for 32 min. The samples were incubated with primary antibodies against iNOS (1:50; PA1–036, Thermo Scientific, Rockford, IL), Arg-1 (1:100; PA5-29645, Thermo Scientific), and CD68 (1:2000; ab955, DAKO, Glostrup, Denmark), respectively. Immunostaining was detected using the DAKO IHC Detection Kit (K1492, DAKO). All IHC slides were counterstained with hematoxylin, dehydrated in ethanol, and cleared in xylene.

### MPO activity assays

The colon (50 mg/mL) was homogenized in phosphate buffer (50 mM, pH 6.0) with 0.5% hexadecyltrimethylammonium bromide (H5882; Sigma, St. Louis, MO) and centrifuged at 12,000 × *g* for 10 min to collect the supernatant. The supernatant (7 μL) was incubated with 200 μL of the reading solution (5 mg *o*–dianisidine [D3252; Sigma], 15 μL 1% H_2_O_2_, 3 ml phosphate buffer, and 27 mL distilled water). The intensity of emitted fluorescence was measured at 450 nm using a fluorescence spectrometer (Sunrise™; TECAN, Männedorf, Switzerland).

### ELISA

The colon was homogenized in PBS containing 1% Triton X-100 and protease inhibitor cocktail and centrifuged at 12,000 × *g* for 10 min at 4 °C. The supernatants were collected and quantitatively analyzed for IL-17 (900K392EK; Peprotech, Rocky Hill, NJ), IL-10 (900K53EK; Peprotech), and TGF-β (MB100B; R&D system, Minneapolis, MN) by ELISA.

### Cytokine antibody array

Colons were homogenized in PBS containing 1% Triton X-100 and protease inhibitor cocktail, centrifuged, and quantified using the Bradford assay. A normalized protein content was analyzed with the Mouse Cytokine Array Panel A (ARY006; R&D system) according to the manufacturer’s instructions. A semi-quantitative analysis of the comparative intensity of the spots was performed with a GS-800 calibrated densitometer image analysis program (GS-800; BIO-RAD, Hercules, CA).

### qRT-PCR

Total RNA was isolated using the Favorprep total RNA mini kit (FABRK001;Vienna, Austria). First-strand cDNA was synthesized from 1 μg of total RNA using the SuperScript^TM^II enzyme (18064-014; Invitrogen). qRT-PCR was performed on an Applied Biosystems 7900HT Fast Real-Time PCR System using the Power SYBR Green PCR Master Mix (326759; Warrington, UK) with the following primers: mβ-actin, GGCTGTATTCCCCTCCATCG (forward) and CCAGTTGGTAACAATGCCATGT (reverse); mMCP-1, CTCACCTGCTGCTACTCATTC (forward) and TTACGGCTCAACTTCACATTCA (reverse); mCXCL9, TGAATTTCCTTGCCACCTTC (forward) and GCCCTGATCTTTCCATTTCA (reverse); mArg-1, CCAGATGTACCAGGATTCTC (forward) and AGCAGGTAGCTGAAGGTCTC (reverse); mIL-10, CATGGGTCTTGGGAAGAGAA (forward) and AACTGGCCACAGTTTTCAGG (reverse); mLIGHT, CTGCATCAACGTCTTGGAGA (forward) and GATACGTCAAGCCCCTCAAG (reverse); mCCL1, CCAGACATTCGGCGGTTG (forward) and CAGCAGCAGGCACATCAG (reverse). The thermal cycling conditions consisted of an initial denaturation step at 95 °C for 5 min, then 35 cycles at 94 °C for 15 s, 55 °C for 15 s, and 72 °C for 15 s, followed by a final extension step at 72 °C for 10 min. The expression levels of the above genes were normalized to that of GAPDH.

### Two-chamber co-culture assays

RAW 264.7 macrophages were placed into the bottom of a 6-well plate. Caco-2 cells were placed on the upper inserts, cultivated to differentiate into polarized monolayers, and then transferred into the same 6-well plate. The inserted membrane, with a pore size of 0.4 μM, was used to allow for the transmission of soluble factors. LPS (1 μg/mL) was applied to RAW 264.7 cells for 4 h, and then the media were changed. Caco-2 cells were then treated with MSC-Ex (30 μg/mL) for 24 h. The levels of IL-17 and IL-10 were evaluated using the RAW 264.7 culture medium. The RAW 264.7 cells were harvested and analyzed using qRT-PCR.

### Immunofluorescence

Caco-2 cells were fixed in 4% formaldehyde and permeabilized with 0.1% TritonX-100. After blocking with 0.5% BSA for 15 min at room temrature the cells were incubated with the E-cadherin antibody (#4065; Cell Signaling technology, Boston, MA) and then with a FITC-conjugated secondary antibody (A120-101F; Bethyl, Montgomery, TX).

### Isolation of peritoneal macrophages

The mouse peritoneal macrophages were collected from the mouse abdomen using a syringe (25 G needle) with 5 mL PBS. The cells were cultured for 4 h to allow attachment in complete RPMI 1640 medium containing 10% FBS and 50 μg/mL penicillin/streptomycin. After removing the floating cells, the attached macrophages were cultured in complete RPMI 1640 medium for 2 days.

### Statistical analysis

All data are expressed as the mean ± s.d. The statistical significance of the differences was evaluated using the non-parametric Wilcoxon rank sum test. Statistical significance was defined as *P* < 0.05.

## Electronic supplementary material

Supplementary Information
