# Supplementary material for: Umbilical cord-derived mesenchymal stem cell extracts reduce colitis in mice by re-polarizing intestinal macrophages
Source: Sci Rep. 2017 Aug 25;7:9412. doi: 10.1038/s41598-017-09827-5 (PMC5573412; doi:10.1038/s41598-017-09827-5)
Supplement: Supplementary file 1 — Supplementary Information [file 41598_2017_9827_MOESM1_ESM.pdf]

Umbilical cord-derived mesenchymal stem cell extracts reduce colitis in mice by re-polarizing intestinal macrophages

Ji-young Song[1], Hyo Jeong Kang[2,3], Joon Seok Hong[4], Chong Jai Kim[2], Jae-Yoon Shim[5], Christopher W Lee[6] & Jene Choi[2*]

[1]Institute for Life Science, University of Ulsan College of Medicine, Asan Medical Center, Seoul, Korea, [2]Department of Pathology, University of Ulsan College of Medicine, Asan Medical Center, Seoul, Korea, [3]Department of Physiology, Asan-Minnesota Institute for Innovating Transplantation, Bio-Medical Institute of Technology, University of Ulsan College of Medicine, Asan Medical Center, Seoul Korea, [4]Department of Obstetrics and Gynecology, Seoul National University Bundang Hospital, Gyeonggi-do, Korea, [5]Department Obstetrics and Gynecology, University of Ulsan College of Medicine, Asan Medical Center, Seoul, Korea, [6]Department of Molecular and Cellular Biology, University of California, Davis, Davis, California, United States of America.

*Correspondence:

Jene Choi, PhD, Department of Pathology, University of Ulsan College of Medicine, Asan Medical Center, 88 Olympic-ro 43-gil, Songpa-gu, Seoul 05505, Korea.

Tel.: +82-2-3010-4555; Fax: +82-2-472-7898; E-mail: jenec@amc.seoul.kr

Supplementary Figure legends

Figure 1S. MSC-Ex and MSC-derived conditioned medium (MSC-CM) have distinct cytokine expression patterns.

Figure 2S. MSC-Ex mediated macrophage M1 to M2 switch is uniquely induced by MSC extracts. Experiments were performed as described in Fig. 6C with MSC-Ex and WI-38 fibroblast extracts.

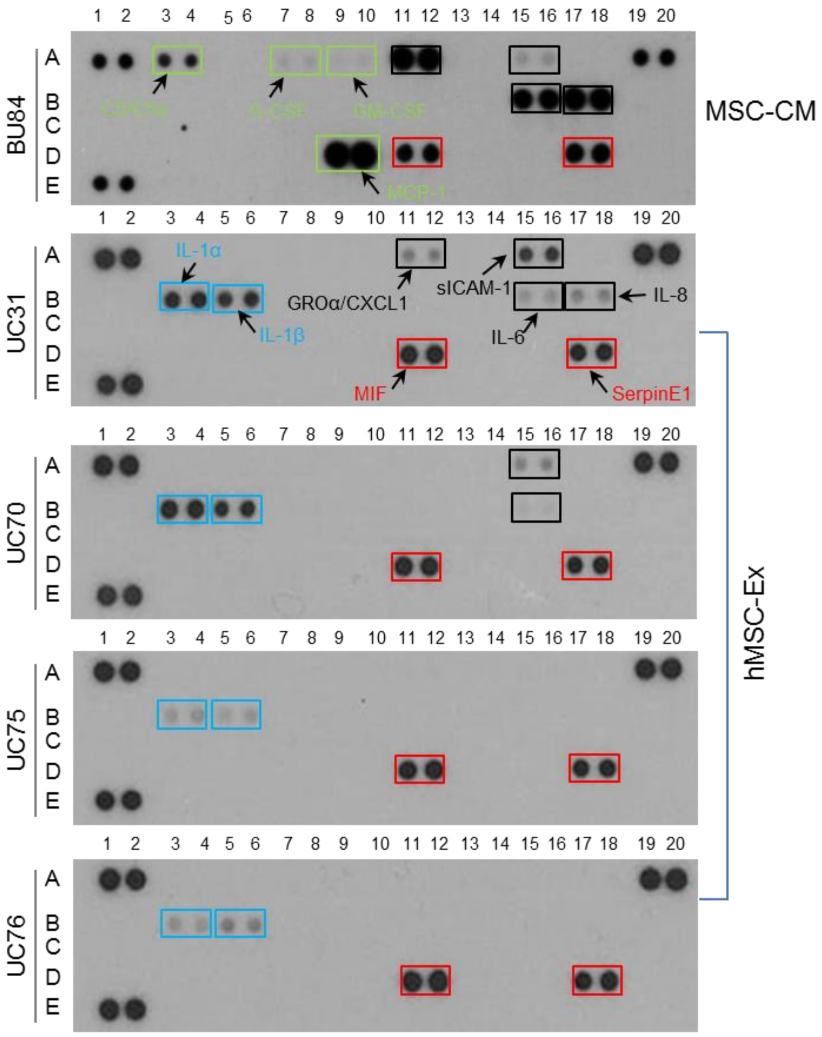

BU84
UC31
UC70
UC75
UC76
MSC-CM
hMSC-Ex
C5/C5a
G-CSF
GM-CSF
MCP-1
IL-1α
IL-1β
GROα/CXCL1
sICAM-1
IL-8
IL-6
MIF
SerpinE1

Figure 1S

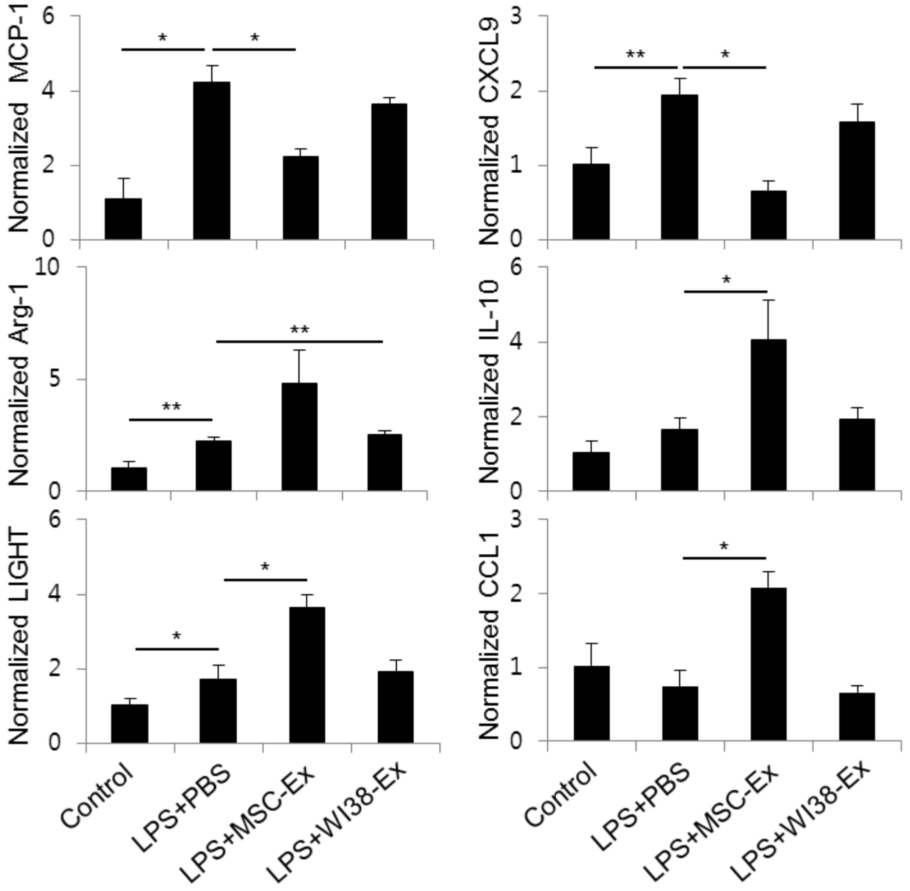

Normalized MCP-1
Normalized CXCL9
Normalized Arg-1
Normalized IL-10
Normalized LIGHT
Normalized CCL1
Control
LPS+PBS
LPS+MSC-Ex
LPS+WI38-Ex

Figure 2S
